# Mice deficient in NKLAM have attenuated inflammatory cytokine production in a Sendai virus pneumonia model

**DOI:** 10.1371/journal.pone.0222802

**Published:** 2019-09-20

**Authors:** Donald W. Lawrence, Laurie P. Shornick, Jacki Kornbluth

**Affiliations:** 1 Department of Pathology, Saint Louis University School of Medicine, Saint Louis, Missouri, United States of America; 2 Department of Biology, Saint Louis University, Saint Louis, Missouri, United States of America; 3 Department of Molecular Microbiology and Immunology, Saint Louis University School of Medicine, Saint Louis, Missouri, United States of America; 4 Veterans Affairs Saint Louis Health Care System, Saint Louis, Missouri, United States of America; University of the Pacific, UNITED STATES

## Abstract

Recent studies have begun to elucidate a role for E3 ubiquitin ligases as important mediators of the innate immune response. Our previous work defined a role for the ubiquitin ligase natural killer lytic-associated molecule (NKLAM/RNF19b) in mouse and human innate immunity. Here, we present novel data describing a role for NKLAM in regulating the immune response to Sendai virus (SeV), a murine model of paramyxoviral pneumonia. NKLAM expression was significantly upregulated by SeV infection. SeV-infected mice that are deficient in NKLAM demonstrated significantly less weight loss than wild type mice. In vivo, Sendai virus replication was attenuated in NKLAM^-/-^ mice. Autophagic flux and the expression of autophagy markers LC3 and p62/SQSTM1 were also less in NKLAM^-/-^ mice. Using flow cytometry, we observed less neutrophils and macrophages in the lungs of NKLAM^-/-^ mice during SeV infection. Additionally, phosphorylation of STAT1 and NFκB p65 was lower in NKLAM^-/-^ than wild type mice. The dysregulated phosphorylation profile of STAT1 and NFκB in NKLAM^-/-^ mice correlated with decreased expression of numerous proinflammatory cytokines that are regulated by STAT1 and/or NFκB. The lack of NKLAM and the resulting attenuated immune response is favorable to NKLAM^-/-^ mice receiving a low dose of SeV; however, at a high dose of virus, NKLAM^-/-^ mice succumbed to the infection faster than wild type mice. In conclusion, our novel results indicate that NKLAM plays a role in regulating the production of pro-inflammatory cytokines during viral infection.

## Introduction

Respiratory infections are a significant cause of morbidity and mortality worldwide. Even though there is a wide variety of distinct respiratory viruses that cause human illness, the clinical presentations for viral respiratory infection are remarkably similar. An emerging hypothesis suggests that it is the host response to the virus that accounts for the pathological effects observed during viral infection [1]. Indeed, the cytokine storm, a dysregulated production of pro-inflammatory cytokines, correlates with lung tissue injury and poor prognosis during influenza infections [2]. Continued research is needed to elucidate mechanisms of inflammation to discover novel therapeutic targets that would allow better control of excess inflammation and lessen host tissue damage.

Sendai virus, a member of the paramyxovirus family, is a natural rodent pathogen and causes neutrophilic bronchiolitis that resembles human paramyxoviral infection. Sendai viral infection is well characterized and leads to expression of inflammatory cytokines that are regulated by immunologically important transcription factors such as STAT1 [3] and NFκB [4]. An emerging body of research demonstrates that protein ubiquitination by ubiquitin ligases of the RING in between RING (RBR) family is one of the processes that play a significant role in regulating cytokine expression.

Natural killer lytic-associated molecule (NKLAM/RNF19b) is a ubiquitin ligase and member of the RBR family of ubiquitin ligases. NKLAM is weakly expressed in resting, unstimulated immune cells; however, upon stimulation with bacterial products or cytokines (e.g. lipopolysaccharide (LPS), interferon gamma (IFNγ), NKLAM expression increases [5]. NKLAM has been localized to NK cell cytolytic granules [6] and macrophage phagosomes [5]. Additionally, we demonstrated that bone-marrow-derived macrophages (BMDM) and neutrophils from NKLAM-deficient mice have a significantly defective bactericidal response [5, 7]. These studies strengthen the role of NKLAM as an important component of the innate immune response. Cytokine production is critical for the successful eradication of invading pathogens during infection. Inflammatory cytokines produced at the sites of infection are responsible for activating resident tissue leukocytes such as macrophages as well as recruiting additional leukocytes from circulation. There is an emerging body of evidence that demonstrates RBR ligases are integral to immune signal transduction and cytokine expression. Two recent studies demonstrated that RBR ligases Parkin and Dorfin/RNF19a function to inhibit immune signaling. Wu et al. showed that NLRP11 recruited RNF19a to catalyze the K48-linked ubiquitination of TRAF6, which ultimately led to TRAF6 degradation, inhibition of Toll-like receptor signaling and increased TNFα, IL-1β and IL-6 expression [8]. Similarly, a study from Xin et al. found that RBR family member Parkin regulated antiviral signaling by targeting TRAF3 for ubiquitin-mediated degradation. In this model, infection of mouse embryonic fibroblasts from Parkin knock out mice with Sendai virus increased inflammatory cytokine expression [9].

In contrast, evidence suggests RBR ligases may also play a positive role in inflammatory cytokine expression. In a recent study from our laboratory we showed that mice lacking NKLAM were defective in the expression of key inflammatory cytokines, such as MCP-1, TNFα, and IFNγ and this defect in cytokine production was associated with a significantly decreased ability to kill *Streptococcus pneumoniae in vivo* [7]. Likewise, the downregulation of RBR ligase Parkin, resulted in decreased IL-6 and MCP-1 expression in response to LPS. The authors also showed that Parkin knockdown did not affect the phosphorylation of IκBα, a component of the canonical NFκB signaling pathway [10]. Similarly, our laboratory showed that the loss of NKLAM did not affect LPS-induced degradation of IκBα, but did affect NFκB nuclear translocation [11]. In a model of acute lung injury, Letsiou et al. found that attenuated IL-6 production and reduced numbers of activated neutrophils in bronchioalveolar lung fluid from Parkin knockout mice was associated with less NFκB phosphorylation [12].

The role of RBR ubiquitin ligases as mediators of the innate immune response has yet to be fully elucidated. At present, data suggest that RBR ligases are capable of both positive and negative regulation of cytokine production depending upon the stimulant and cell type used. Understanding precisely how RBR ligases are involved in regulating the immune response to pathogens may lead to the identification of novel therapeutic targets.

In this study, we examined the role of E3 ubiquitin ligase NKLAM during respiratory viral infection. Using a mouse model of paramyxoviral infection we show that NKLAM is a positive effector of the innate immune response. Our data demonstrate that NKLAM plays a role in regulating the expression of inflammatory cytokines and chemokines. In NKLAM^-/-^ mice, the reduced cytokine expression during SeV infection was associated with less leukocyte infiltration into the lungs and less lung inflammation.

## Materials and methods

### Ethics statement

All mouse experiments were reviewed and approved by the Institutional Animal Care and Use Committee at Saint Louis University (Protocol 1287 and 1586) and the Saint Louis Veterans Affairs Healthcare System (Protocol 1108–3115 and 1701–522). This study was carried out in strict accordance with the recommendations in the Guide for the Care and Use of Laboratory Animals of the National Institutes of Health.

### NKLAM^-/-^ mouse generation

NKLAM^-/-^ mice were generated in our laboratory [13]. Briefly, 129Sv/Ev (inGenious Targeting Laboratory) embryonic stem cells containing the NKLAM targeting construct were microinjected into C57BL/6 blastocysts. This construct generates deletion of the RBR domains and most of the transmembrane domains. The offspring of chimeric males and C57BL/6 mice were screened for germline transmission of the NKLAM^-/-^ allele. Heterozygotes were crossed to obtain homozygous offspring. Mice were backcrossed for 11 generations onto a C57BL/6 background. We maintain colonies of both NKLAM^-/-^ and C57BL/6 mice.

### Bone marrow-derived macrophage generation

Euthanized mice were sprayed with 70% ethanol and the tibias and femurs were removed and flushed with DMEM. The bone marrow was resuspended in BM20 media (DMEM with 20% L929-cell conditioned media, 20% fetal bovine serum, 1 mM sodium pyruvate, 100 U/mL streptomycin, 100 U/mL penicillin, and 2 mM L-glutamine). After 7 days of culture in non-tissue culture dishes, the media was changed on day 3.

### Mouse bronchiolitis model

Mice were anesthetized by intraperitoneal injection (100 μL/10g body weight) of xylazine (1 mg/mL) and ketamine (7.5 mg/mL) and 30 μL of sterile PBS or SeV (strain 52; ATCC) was administered to the nares of each mouse. Following infection, the mice were monitored for signs of distress. At the required timepoints, the mice were euthanized with CO_2_. The lungs and trachea were removed and homogenized for 30s in 1 ml or 250 μL sterile PBS, respectively, using a tissuemiser (Fisher Scientific). For the high-dose survival experiments, WT and NKLAM^-/-^ mice were infected intranasally with 5 x 10^3^ or 5 x 10^4^ SeV pfu/gram body weight and monitored several times a day for signs of severe sickness (e.g. lethargy, hypothermia, piloerection, inability to maintain upright posture). Extremely ill mice were euthanized immediately by CO_2_ asphyxiation and scored dead for the purposes of the experiment.

### Immunoblotting

Mouse proteins were separated by SDS-PAGE and transferred to PVDF membrane. After blocking in TBS-T with 1% BSA in (TBS-T), the blots were incubated with primary antibody (1:1000 dilution) overnight. The rabbit monoclonal antibodies for LC3 (#12741), STAT1 (#9172), pSTAT1-Tyr701 (#9167), NFκB phospho-p65 (Ser536) (#3033), p62/SQSTM1 (#23214) and NFκB p65 (#8242) were obtained from Cell Signaling. The anti-NKLAM antibody has been described previously [6]. The blots were washed 3 times in TBS-T and incubated with horse radish peroxidase-conjugated secondary antibodies. The images were captured with a BioRad Chemidoc XRS+ imager. Protein normalization was performed using β-actin Western blot or with 2,2,2-trichloroethanol (TCE). Briefly, TCE (54 mM final concentration) was added to the polyacrylamide gel solution prior to polymerization. TCE binds to tryptophan residues and becomes fluorescent when exposed to UV light [14], allowing the visualization of the total protein in each lane. For normalization, the band intensity for a protein of interest was divided by the intensity of the TCE signal in the corresponding lane. For phosphoprotein analysis (pSTAT1 and pp65), the band intensity of the phosphorylated form of the protein was divided by the band intensity of the total protein.

### Lung histological analysis

For H&E staining, the mice were euthanized by CO_2_ asphyxiation and the lungs were inflated with 10% formalin. Lungs were removed and after ethanol dehydration, embedded in paraffin, sectioned and stained with H&E. Histological assessment was performed visually by two independent investigators. Original magnification: x50.

### Immunofluorescence

WT and NKLAM^-/-^ BMDM were plated on 18mm glass coverslips at a density of 3 x 10^5^ cells per well. After treatment with rapamycin (1 μM for 18 hr) the monolayers were quickly washed in ice-cold PBS and fixed for 20 min in -20°C methanol. The cells were washed 2 times, 5 min each with PBS and then blocked with 5% normal goat serum, 0.3% TX-100 in PBS for 60 min at room temperature. The fixed monolayers were stained overnight with anti-LC3 (1:200, Cell Signaling #3868). Image processing was minimal and was performed equally on all images. LC3 puncta were enumerated using ImageJ v.1.49. At least 250 cells were counted per each genotype.

### Quantitative PCR

Total RNA was isolated using the Qiagen RNeasy kit. One hundred nanograms of RNA was used for cDNA synthesis using the Taqman reverse transcription kit (Applied Biosystems). Quantitative PCR was performed with Syber Select Master Mix (Applied Biosystems). 18S was used for normalization. For data presentation, the mean ΔΔCt values for each gene were calculated by subtracting the mean ΔCt value of the reference gene (18S) from the mean ΔCt value of the target gene. Data were calculated as 2^ΔΔCt^ and expressed as fold change relative to untreated cells or mice given PBS intranasally.

### Lung cytokine profile

Lung cytokine expression was determined using cytokine arrays purchased from Raybiotech (catalog # AAM-CYT-3). Mice were infected with SeV (500 pfu/gram body weight) for 3 or 7 days and the lungs were excised and homogenized in PBS supplemented with protease inhibitors. Cleared lung homogenate protein (500 μg) was used for cytokine determination. The membranes were developed and quantified using a BioRad Chemidoc XRS+ imager. The intensities were normalized to internal positive controls.

### Flow cytometry

Infected mouse lungs (500 SeV pfu/gram body weight) were mechanically homogenized using the gentleMACS tissue dissociator (Miltenyi). The resulting cell suspension was passed through a 70 μm mesh filter then washed once in ice-cold PBS. Cells (300,000) were incubated with Fc block for 20 min on ice then stained with fluorescent-labeled primary antibodies for 30 min on ice in the dark. CD45-PerCP, NK1.1-APC, Gr-1-FITC and CD3-Alexa Fluor 700 antibodies were purchased from BD Biosciences. The F4/80-efluor 450 was purchased from eBioscience and the CD11b-PE was purchased from Invitrogen. Cells were washed once in ice-cold staining buffer (PBS + 1% BSA and 0.1% sodium azide) then fixed with 1% formaldehyde. Total number of specific leukocyte population per lung was calculated as follows: # of specific leukocyte population = total lung cell number x %gated cells x %single cells x %CD45^+^ cells x %specific leukocyte population. Flow cytometric data were analyzed with FlowJo software (Treestar, Ashland, OR).

### Lung fibroblast isolation

Wild type or NKLAM^-/-^ mouse lungs were mechanically homogenized using the gentleMACS tissue dissociator (Miltenyi). The resulting cell suspension was passed through a 70 μm mesh filter then washed twice in ice-cold PBS. The single cell suspension was resuspended in DMEM plus 10% FBS and plated in 100 mm petri dishes for 48h. The plates were rinsed to remove non-adherent cells and maintained in culture until confluent monolayers of fibroblasts were evident.

### Mouse tracheal epithelial cell (mTEC) isolation

Mouse tracheal epithelial cells were isolated using the method of You et al. [15]. Mice were euthanized with CO_2_ and sprayed with 70% ethanol. The tracheas were excised and collected in ice-cold DMEM. Tracheas were washed with media then incubated in Ham's F-12 pen-strep containing 1.5 mg/ml pronase for 18 h at 4°C. The tube was then put on ice and FBS was added to a final concentration of 10%. The tracheas were inverted several times to release the epithelial cells. The cells were collected by centrifugation then resuspended in 200 μl/trachea of Ham's F-12 pen-strep containing 0.5 mg/ml crude pancreatic DNase I (Sigma-Aldrich) and 10 mg/ml BSA. The cells were then incubated on ice for 5 min, collected by centrifugation and then resuspended in mTEC basic media with 10% FBS. This crude tracheal isolate was used to examine NKLAM expression. To assess cytokine production, the crude tracheal suspension was incubated in 60 mm tissue culture plates for 3h to adhere fibroblasts, then nonadherent cells were collected by centrifugation and resuspended in 100–200 μl mTEC/Plus per trachea. The epithelial cells were plated in transwell inserts until confluent. The medium was then removed to initiate mucociliary differentiation. The monolayers were then infected with SeV at MOI 0.1 for 1h to promote virus adsorption then cultured for 72h. Total RNA was isolated with PureLink RNA mini kit (Invitrogen).

### Statistical analysis

Statistical analyses (two-tailed, unpaired Student’s *t*-test, two-factor ANOVA or log-rank test) were performed using Microsoft Excel or GraphPad software. A p value of 0.05 or less was considered statistically significant.

## Results

### Sendai infection induces NKLAM expression in mice

**Fig 1:**
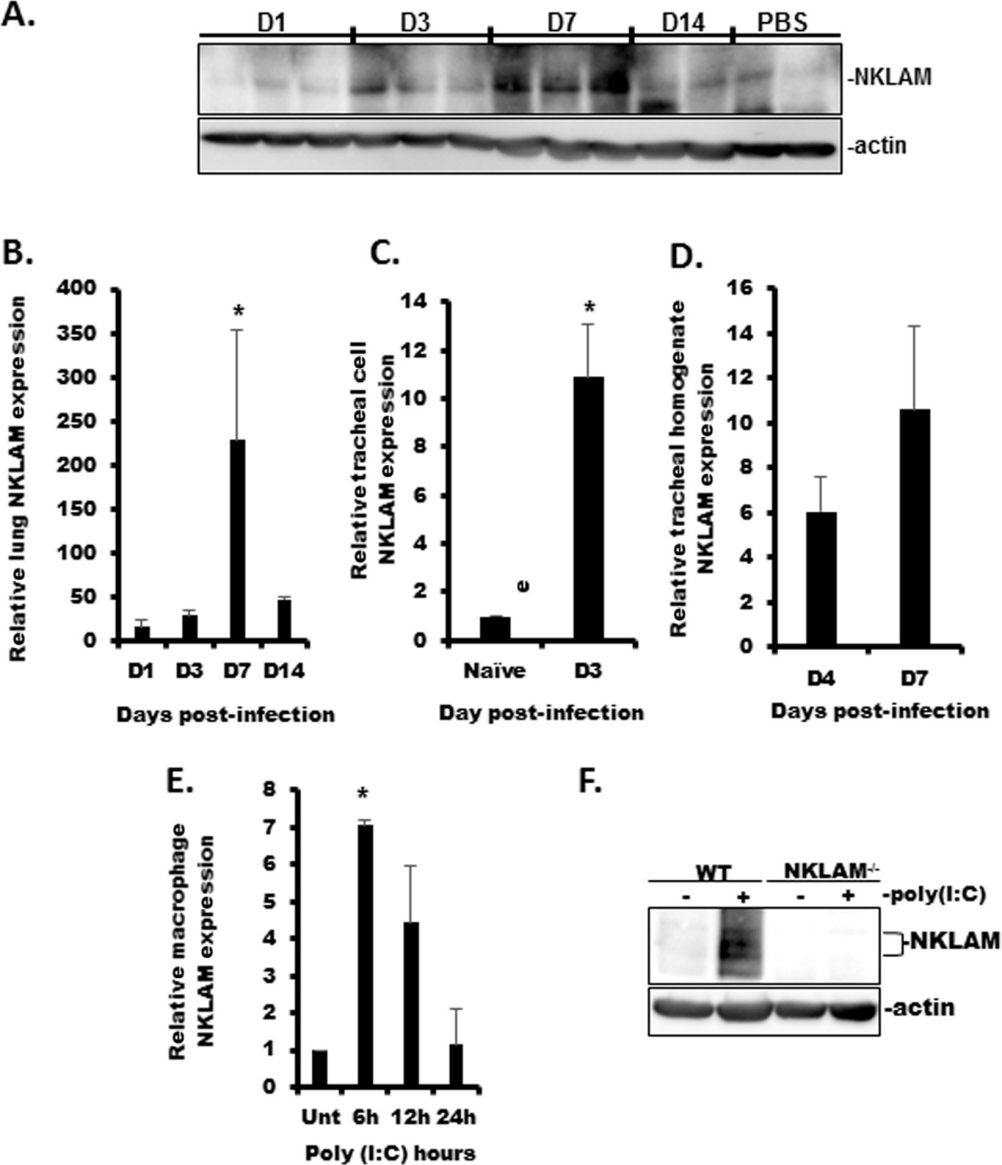
NKLAM expression is increased during SeV infection. A) NKLAM protein expression was assessed by Western blot. Each lane represents a single mouse. B) NKLAM protein expression by qPCR using lung homogenate during SeV infection. C) NKLAM mRNA expression in isolated SeV-infected mouse tracheal epithelial cells was determined by qPCR. D) NKLAM expression in infected (D4 and D7) tracheal homogenate was determined by qPCR. E) WT BMDM were treated with 100 μg/ml poly(I:C) for times indicated and NKLAM expression was assessed by qPCR. The mRNA levels (mean ± SD) are expressed relative to PBS-treated mice or unstimulated BMDM. n = 3 mice per group; * p < 0.05. F) WT and NKLAM^-/-^ BMDM were treated with 100 μg/ml poly(I:C) for 18h and NKLAM expression was determined by Western blot. Data are representative of two independent experiments.

NKLAM is an interferon-stimulated gene (ISG) as its expression is increased by IFNβ [16] and IFNγ [5] treatment, but the effect of viral infection or viral nucleic acid mimetics on NKLAM expression is not known. To this end, we examined the expression kinetics of NKLAM in WT lungs and trachea and isolated tracheal cells during SeV infection. Lung homogenate was used to examine the expression of NKLAM by Western blot (Fig 1A) and qPCR (Fig 1B) during SeV infection. By Western blot, NKLAM was not significantly expressed in unstimulated (PBS) lung tissue nor lung tissue from day 1 post-infection. By day 3, the levels of NKLAM protein were increased and were maximal at day 7. Using qPCR, we found that increased NKLAM expression was observed as early as day 1 as compared to PBS control. NKLAM expression was maximal at day 7 then decreased at day 14. We also examined NKLAM expression in isolated mouse tracheal epithelial cells by qPCR. Uninfected tracheal epithelial cells expressed a minimal amount of NKLAM mRNA. Infection with SeV significantly increased NKLAM expression at day 3 post-infection in the airway cells (Fig 1C). NKLAM expression was also significantly upregulated in tracheal homogenates at D4 and D7 (Fig 1D). Additionally, we observed that *in vitro* treatment with viral nucleic acid mimetic poly(I:C) increased NKLAM mRNA and protein in BMDM (Fig 1E and 1F).

### NKLAM^-/-^ mice lose less body weight and regain weight faster than WT mice during infection with SeV

**Fig 2:**
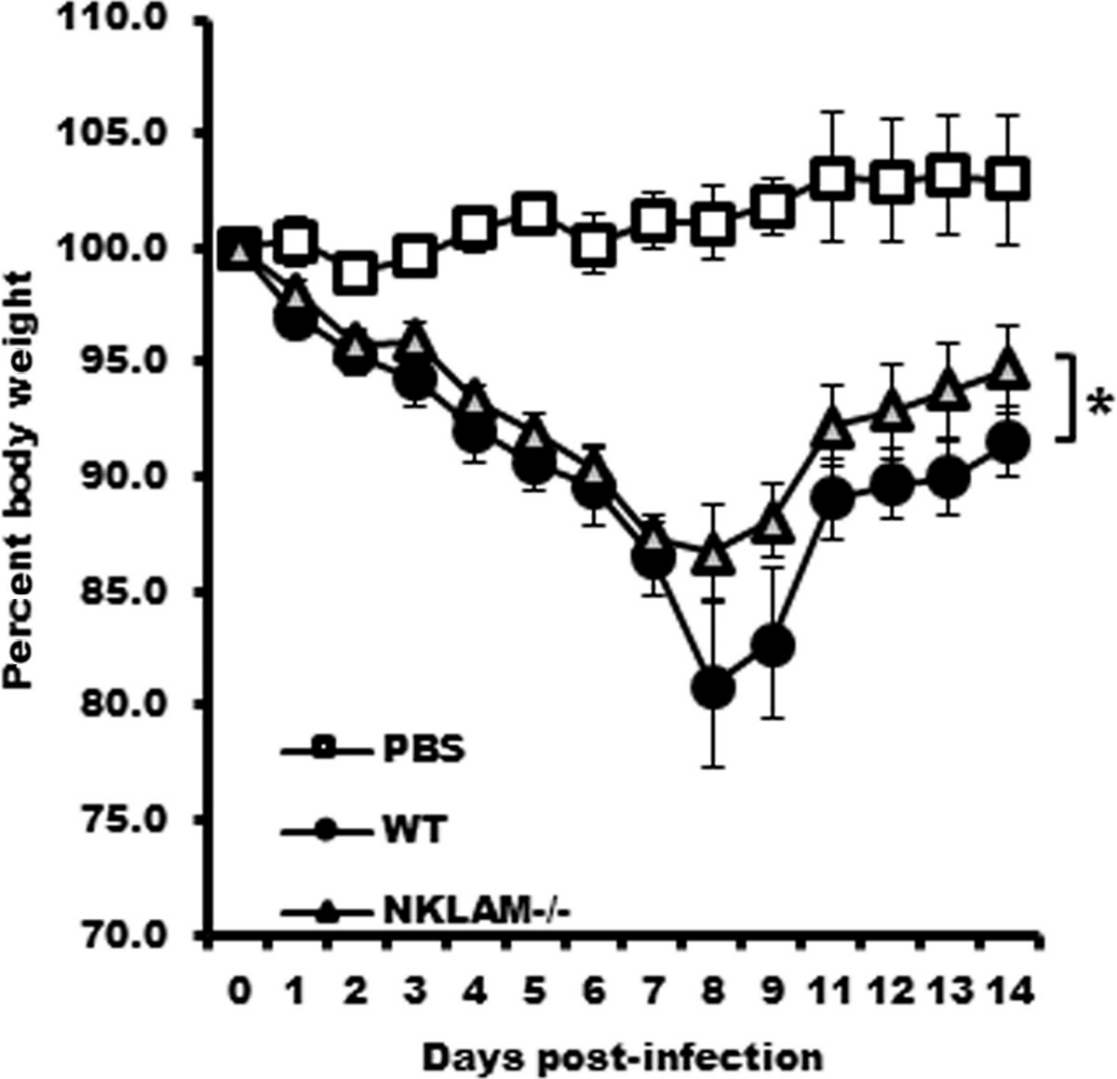
NKLAM^-/-^ mice experience less weight loss during SeV infection. All mice were infected with SeV (500 pfu/gram body weight) and their weight was monitored daily for 14 days. PBS (30 μL) was administered to uninfected control mice. n = 10 mice per group; p = 0.0004, Two-factor ANOVA.

Weight loss is a defining characteristic of murine viral pneumonia. We assessed total body weight loss during SeV infection in WT and NKLAM^-/-^ mice. As shown in Fig 2, both groups of mice infected with SeV (500 pfu/gram body weight) began to lose weight at day 1 and their weight continued to decline for 7 days with no discernable difference between groups. After day 7 the WT mice continued to lose weight while the rate of weight loss slowed in the NKLAM^-/-^ group from day 7 to day 8, suggesting that the NKLAM^-/-^ mice resolve inflammation faster than the WT mice. There is significantly more weight loss in WT mice overall. After day 8, the WT mice start regaining weight and both groups showed positive weight gain for the remainder of the study.

### SeV viral expression in WT and NKLAM^-/-^ tissues

**Fig 3:**
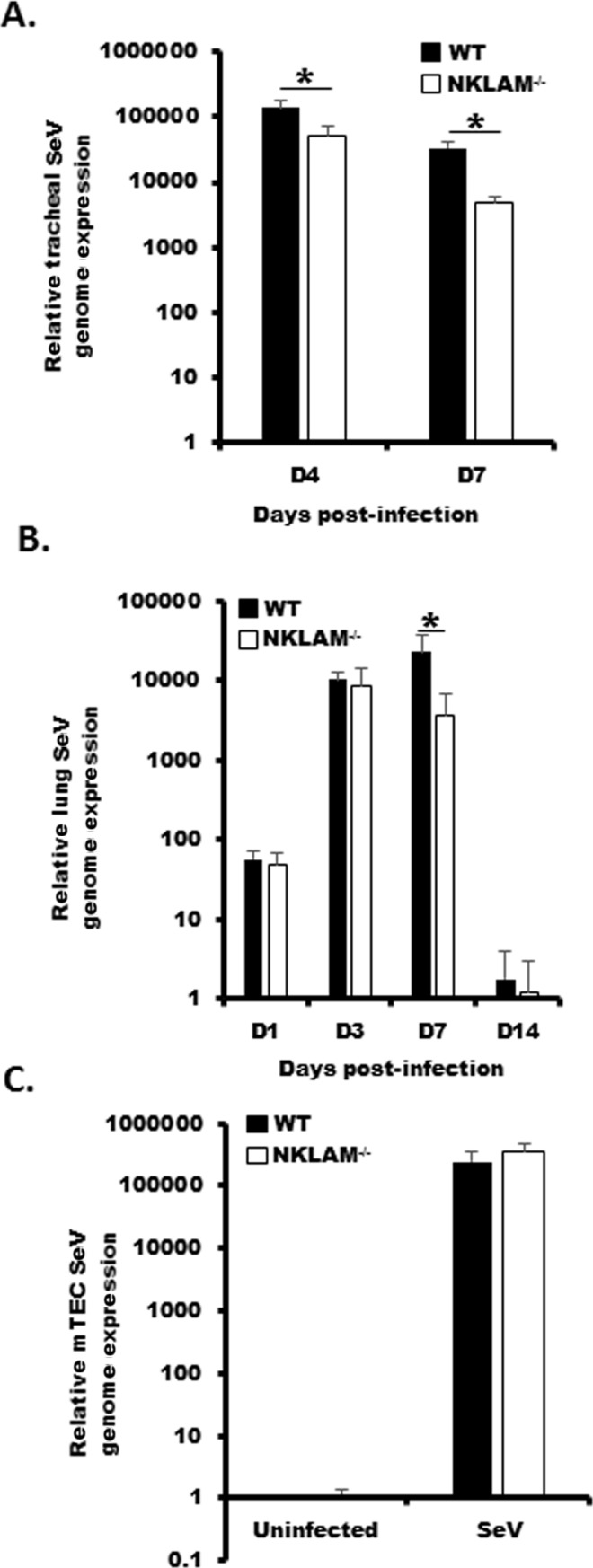
NKLAM^-/-^ mice have a significantly lower viral load during SeV infection. Mice were infected with 500 pfu SeV/gram body weight and homogenized tracheal (A) or lung tissue (B) was used to determine SeV viral load by qPCR. * p < 0.05; n = 3–8 mice per group; comparing NKLAM^-/-^ and WT mice at each day post-infection. C) mTEC were infected with SeV at an MOI of 0.1 for 72h and the SeV genome expression was determined by qPCR. Data represents three replicates.

Our next set of experiments compared the viral load in WT and NKLAM^-/-^ trachea, lungs and mTEC during SeV infection. Using mRNA isolated from SeV infected tracheal homogenates, we unexpectedly found that NKLAM^-/-^ mice had significantly less SeV genome expression at day 4 and day 7 as compared to WT mice (Fig 3A). The levels of SeV genome expression within the trachea decreased over time as the infection progressed into the lung. As shown in Fig 3B, the viral load in the lungs of WT and NKLAM^-/-^ mice was similar up to day 3 post-infection. However, at day 7, the relative SeV genome expression increased over 3-fold in the lungs of WT mice, while it decreased by a similar extent in NKLAM^-/-^ mice. In cultured mTEC the expression of SeV was similar between WT and NKLAM^-/-^ after 72h of infection with SeV (Fig 3C).

### Attenuated STAT1 and NFκB phosphorylation in the lungs of SeV-infected NKLAM^-/-^mice

**Fig 4:**
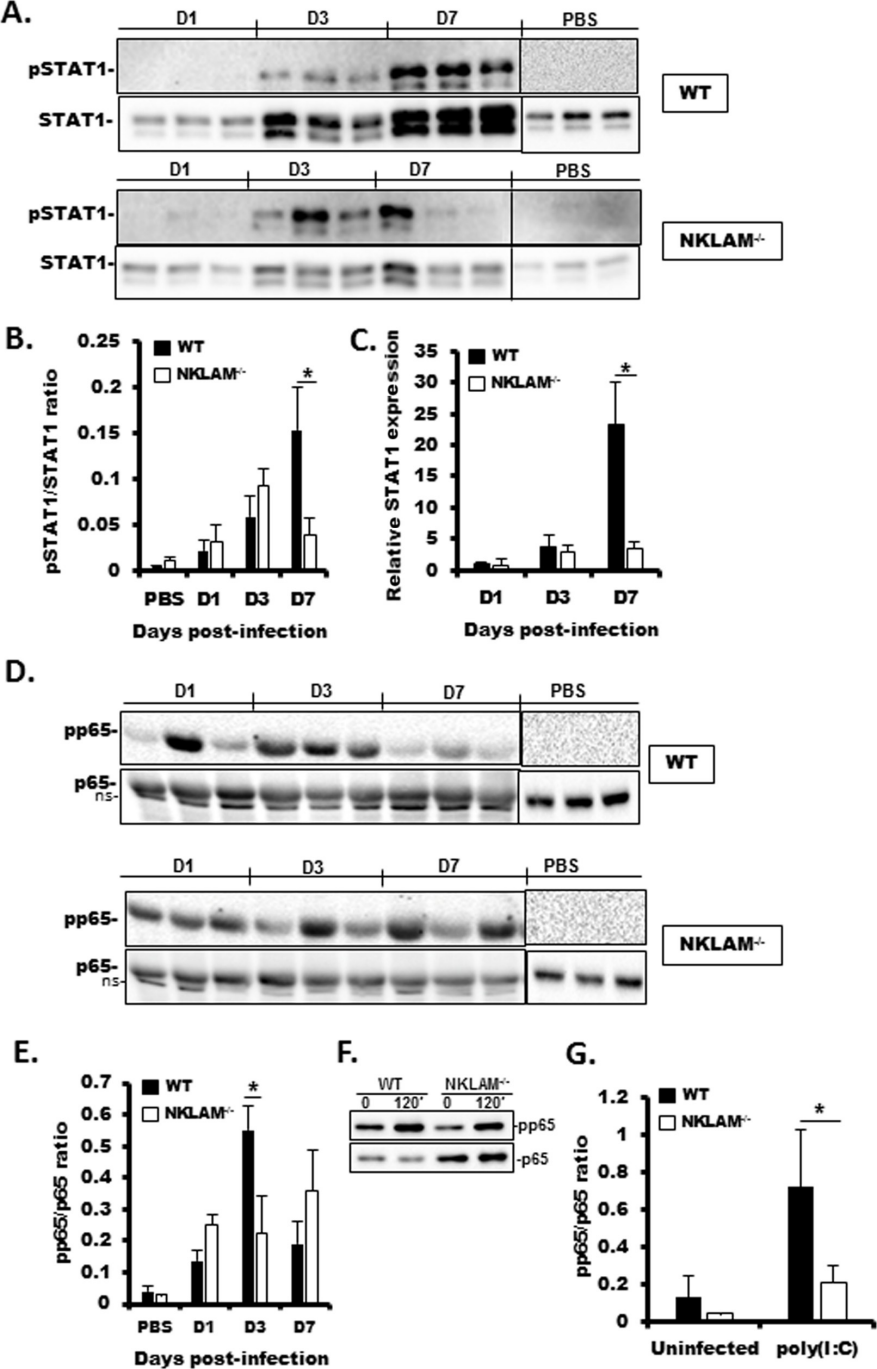
SeV-induced STAT1 protein expression and STAT1 and NFκB p65 phosphorylation are lower in NKLAM^-/-^ than in WT mice. WT and NKLAM^-/-^ mice were infected with 500 pfu SeV/gram body weight. A) Equal amounts (20 μg) of lung homogenate protein were then probed for pSTAT1(701) and STAT1. B) Graphical depiction of pSTAT1/STAT1 densitometric ratio from A. Quantitative PCR was used to determine the relative expression of STAT1 in infected lungs (C). The mRNA levels (mean ±SD) are expressed relative to PBS-treated mice. * p < 0.05; n = 3 mice per group. D) Equal amounts of lung homogenate protein were probed for phospho-NFκB p65 (S536) and p65. E) Graphical depiction pp65/p65 densitometric ratio of D. Each lane of Western blot data represents a single mouse. * p < 0.05; comparing NKLAM^-/-^ and WT mice at each day post-infection. n = 3 mice per group. ns; non-specific band. F) Isolated lung fibroblasts were treated with 100 μg/ml poly(I:C) for 120 min. Whole cell lysates were immunoblotted for pp65 and p65. G) Graphical representation of pp65/p65 densitometric ratio from F. Data are representative of 3 independent experiments. * p ≤ 0.05.

Recent studies from our laboratory demonstrated that STAT1 tyrosine phosphorylation is lower in NKLAM^-/-^ than in WT mice upon bacterial infection [7]. We found that infection with SeV induced the phosphorylation of tyrosine 701 in STAT1 in the lungs of both WT and NKLAM^-/-^ mice beginning at day 3 post-infection. However, phospho-STAT1 levels were significantly higher in lungs of WT mice than in NKLAM^-/-^ mice at day 7 (Fig 4A and 4B). STAT1 is an ISG and expression of STAT1 increases during infection with SeV [3]. Using qPCR, we show that STAT1 mRNA expression increased at day 3 and was maximal in WT mice at day 7 post-infection. NKLAM^-/-^ mice expressed significantly less STAT1 protein at day 7 as compared to WT mice (Fig 4C). NFκB is an important regulator of the immune response and is responsible for regulating the expression of gene products important for a functional immune response. It is known that SeV infection induces NFκB p65 phosphorylation at serine 536 [4, 17]. Studies from our laboratory have demonstrated that NKLAM positively affects the nuclear translocation and phosphorylation of NFκB subunit p65 [11]. Thus, our next experiment was to examine the phosphorylation state of NFκB p65 in WT and NKLAM^-/-^ mouse lungs during SeV infection. As shown in Fig 4D and 4E, there was significantly less NFκB p65 phosphorylation at day 3 post-infection in NKLAM^-/-^ mouse lungs in comparison to lungs of WT mice. Additionally, pp65 phosphorylation was also significantly less in lung fibroblasts isolated from NKLAM^-/-^ mice (Fig 4F and 4G). Collectively, the observed effect of NKLAM on p65 phosphorylation during SeV infection is consistent with our previous data that showed diminished p65 phosphorylation in NKLAM^-/-^ macrophages and further supports our underlying hypothesis that NKLAM plays a positive role in NFκB signaling [11].

### Inflammatory cytokines are reduced in the lungs of NKLAM^-/-^ mice infected with SeV

**Fig 5:**
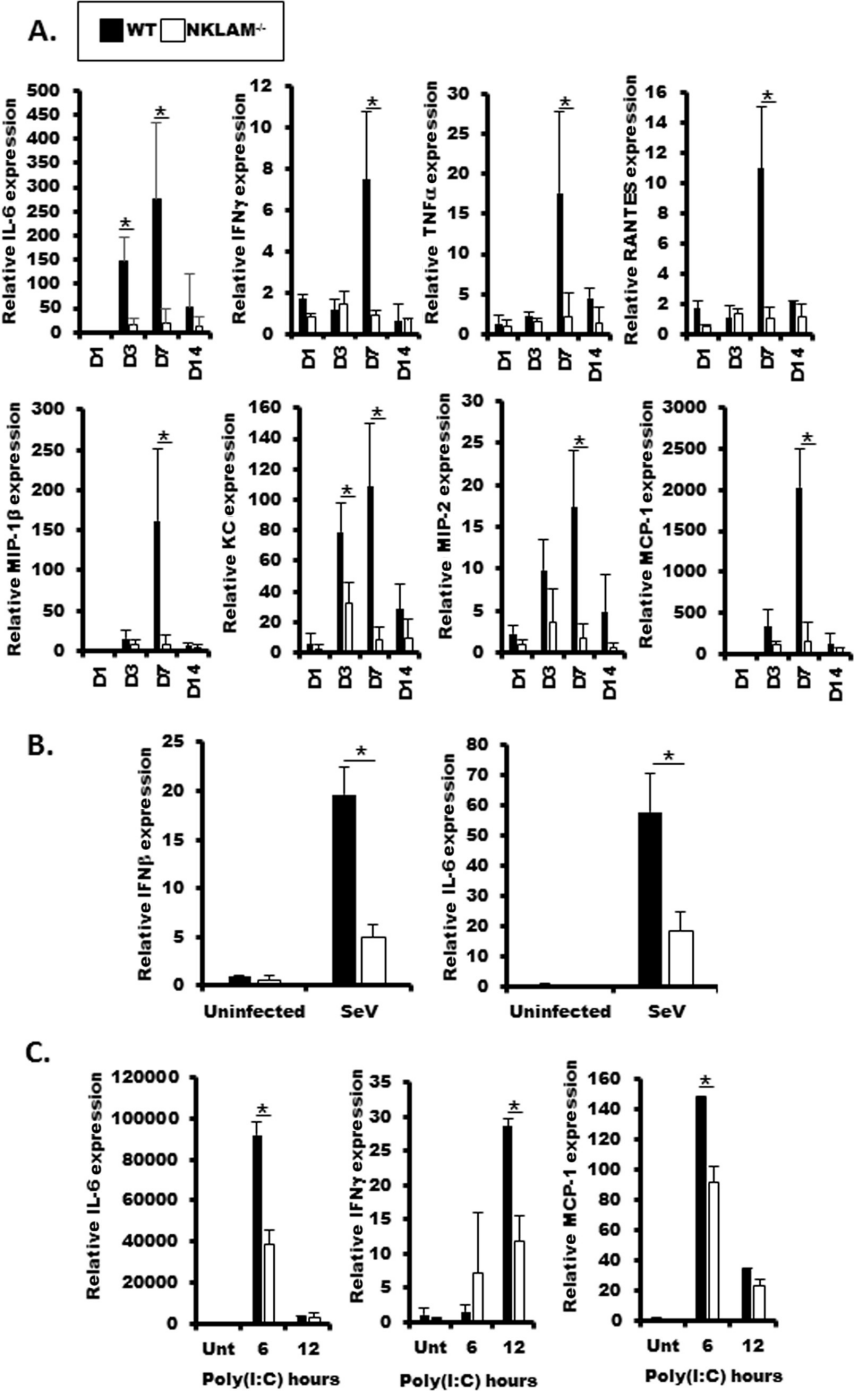
Cytokine expression is less in NKLAM^-/-^ mouse lung homogenate and NKLAM^-/-^ BMDM. A) RNA isolated from lung homogenate from SeV-infected lungs was used to determine cytokine expression by qPCR. B) Isolated mTEC were incubated with SeV MOI 0.1 for 72h and IFNβ and IL-6 expression was determined by qPCR. C) BMDM were treated with 100 μg/ml poly(I:C) for 6 or 12 hours and cytokine expression was determined by qPCR. The mRNA levels (mean ± SD) are expressed relative to PBS-treated mice (n = 3 mice per group) or untreated mTEC or BMDM; * p < 0.05. Statistical comparisons are made between WT and NKLAM^-/-^ mice.

Our previous studies demonstrate that NKLAM is involved in regulating the expression of key inflammatory cytokines in a mouse model of *S*. *pneumoniae*-induced pneumonia [7]. In our next set of experiments, we examined cytokine levels in lung homogenates from WT and NKLAM^-/-^ mice at key timepoints during SeV infection. We used a cytokine protein array to measure the expression of 62 cytokines from the lungs of SeV-infected WT and NKLAM^-/-^ mice at day 3 and day 7 post-infection. The results are shown in Table 1. We listed cytokines that were at least 1.5 times greater in the lungs of infected WT mice than NKLAM^-/-^ mice at day 3 or day 7. Among this group of cytokines were both pro- (IL-1α, IL-2, IL-6, IL-17A) and anti-inflammatory cytokines (IL-10, IL-13). In addition, key chemokines such as KC (murine IL-8 homolog), MCP-1, RANTES, and MIP-2 were also expressed to a greater degree in WT mice compared to NKLAM^-/-^ mice. Several of the genes listed in Table 1 are regulated by transcription factors STAT1 (#), NFκB (*) or both. Interestingly, IL-6, a potent proinflammatory cytokine, was expressed 1.7 times greater in the lungs of WT than NKLAM^-/-^ mice at day 3 post-infection. By day 7, IL-6 in WT lungs was nearly 23-fold greater than in NKLAM^-/-^ lungs. We next performed qPCR on lung homogenate to evaluate the expression of several key cytokines that were shown to be upregulated by cytokine array. As shown in Fig 5A, chemokines critical for macrophage and neutrophil recruitment were higher in WT mice as compared to NKLAM^-/-^ mice. The expression of chemokines MIP-2, MCP-1, MIP-1β and RANTES was significantly higher in WT mice at day 7 as compared to NKLAM^-/-^ mice. KC, a potent murine neutrophil chemoattractant was significantly elevated in WT mice at day 3 and day 7. IL-6 was also significantly elevated in WT mice at day 3 and day 7. Additionally, we show that IFNγ and TNFα are significantly elevated in WT mice at day 7. Using isolated tracheal epithelial cells (mTEC), we found the levels of IFNβ and IL-6 were also lower NKLAM^-/-^ cells (Fig 5B). We also examined cytokine expression in BMDM isolated from WT and NKLAM^-/-^ mice stimulated with viral nucleic acid mimetic poly(I:C). In agreement with our *in vivo* studies, IL-6, IFNγ and MCP-1 levels were significantly lower in BMDM from NKLAM^-/-^ mice (Fig 5C). Collectively, these data strengthen our overall hypothesis that NKLAM is an important regulator of the innate immune system, through expression of inflammatory chemokines and cytokines.

**Table 1:** Cytokine and chemokine expression in SeV-infected WT and NKLAM^-/-^ lungs.

	Day 3 post-infection			Day 7 post-infection			
	WT	NKLAM^-/-^	fold	WT	NKLAM^-/-^	fold	Ref.
IL6*	42571	24810	1.70	90102	3949	22.80	[18]
SFD-1α*	4126	9744	0.42	58464	15421	3.80	[19]
SCF*	1242	2354	0.53	9341	2739	3.40	[18]
**IL-5**	4874	5000	0.97	4437	1337	3.30	
Eotaxin-1*	4712	3675	1.30	15884	4954	3.20	[18]
IL-13*	8866	5845	1.50	6497	2413	2.70	[18]
BLC*	24932	18083	1.40	79363	29196	2.70	[20]
**CD30 ligand**	1804	1580	1.10	11559	4399	2.60	
MIP-2*#	26028	26951	0.97	57675	22087	2.60	[18, 21]
IL-10*	2414	2225	1.10	8955	3701	2.40	[22]
**Axl**	12483	11902	1.00	34232	15745	2.20	
KC*#	57174	67877	0.84	38993	19373	2.00	[18, 21]
RANTES*#	94880	107037	0.89	243227	146547	1.70	[23, 24]
CTACK*	34421	31160	1.10	17155	10292	1.70	[25]
MCP-1*#	222550	195098	1.10	771719	441773	1.70	[18, 26]
IL-1A*	20562	17551	1.20	14037	9278	1.50	[18]
GM-CSF*	17697	7431	2.40	6855	5203	1.30	[27]
**IL-12 p40/p70**	23765	28290	0.84	110295	94636	1.20	
**TECK**	2946	790	3.70	5840	14774	0.40	
IFNγ*#	n.d.	3977	-	8780	n.d.	-	[18, 26]
IL-17A*	1213	n.d.	-	4204	n.d.	-	[28]
IL-2*	2055	8704	4.20	1833	n.d.	-	[18]
**IL-9**	10793	11616	0.93	n.d.	15074	-	
MIP-1α*	n.d.	2845	-	8828	n.d.	-	[18]
CRG-2/IP-10*	3154	158	20.0	555	n.d.	-	[29]
G-CSF*	1332	n.d.	-	n.d.	n.d.	-	[30]
Leptin*	n.d.	4185	-	5962	n.d.	-	[31]
**IL-3**	2332	5579	2.40	3871	n.d.	-	
**IL-3R beta**	13557	7155	1.90	4034	5353	0.80	

Lung homogenate (500 μg) was pooled from WT or NKLAM^-/-^ (n = 3 mice per genotype) mice at day 3 and day 7 post-infection and incubated with cytokine array membranes for determination of cytokine expression. The densities were normalized to internal positive controls. The densitometric value for each gene is the average of two spots.

(*) NFκB regulated genes

(#) STAT1 regulated genes. n.d., not detected.

### Lack of NKLAM^-/-^ affects leukocyte recruitment into the lung during SeV infection

**Fig 6:**
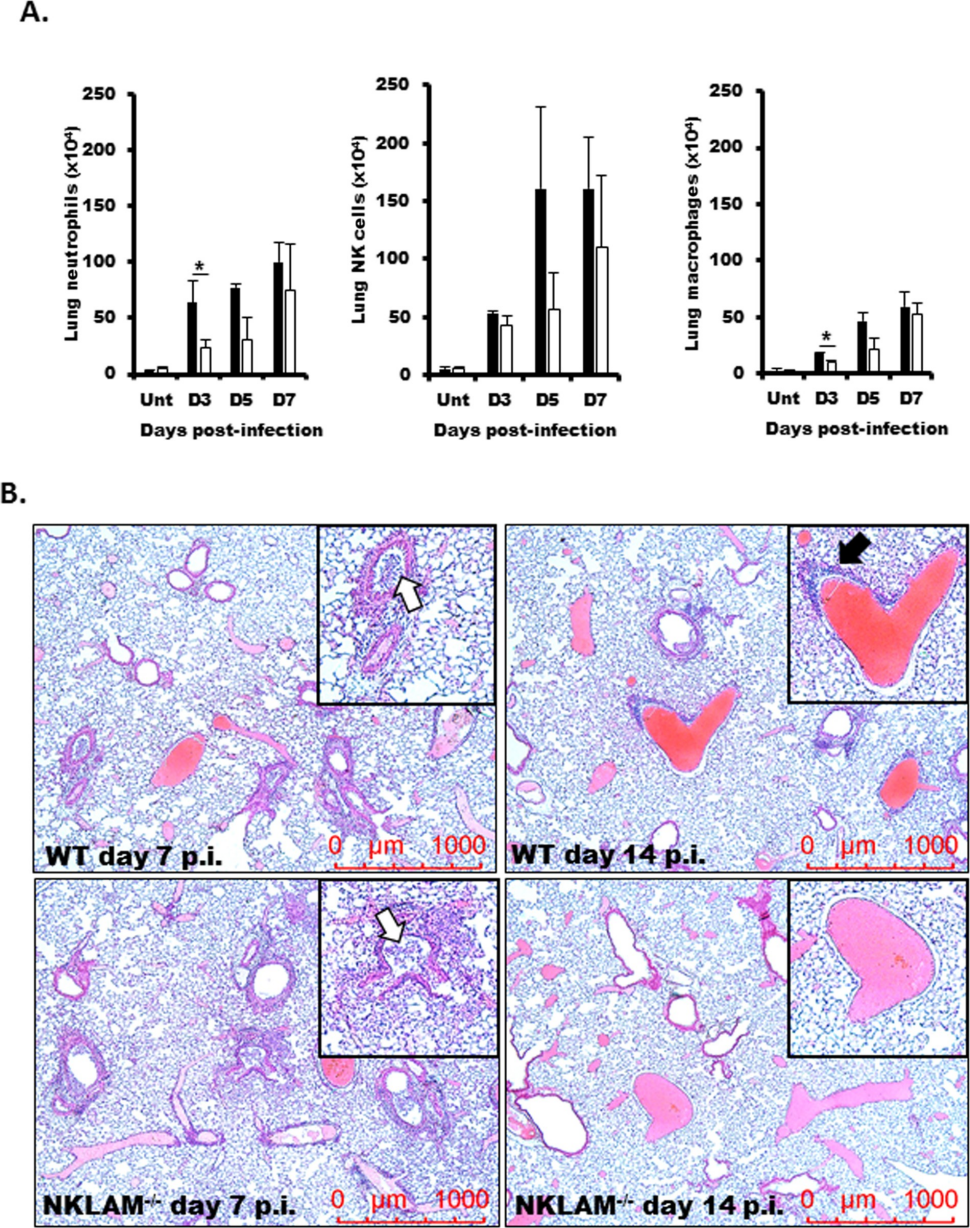
Examination of leukocyte infiltration into the lungs during SeV infection. WT and NKLAM^-/-^ mice were uninfected (Unt) or infected with 500 pfu SeV/gram body weight. A) Lungs were dissociated into single cell suspensions were isolated at day 3, 5, and 7 post-infection and stained for CD45, CD3, NK1.1, CD11b, Gr-1 and F4/80. CD45^+^ cells were gated and the number of CD11b^+^/Gr-1^+^, CD3^-^/NK1.1^+^ or CD11b^+^/F4/80^+^ cells was determined; * p < 0.05; n = 3 mice per group, comparing NKLAM^-/-^ and WT mice. B) Lungs from post-infection (p.i.) day 7 and 14 were formalin-fixed, embedded in paraffin and sections were stained with H&E. n = 2 mice per condition. Original magnification x50.

We next assessed specific leukocyte populations by flow cytometry. Single cell suspensions from uninfected and SeV-infected lungs at days 3, 5, and 7 were used to assess the total numbers of neutrophils, macrophage and NK cells in WT and NKLAM^-/-^ lungs. As shown in Fig 6A, we found that NKLAM^-/-^ mice had significantly lower numbers of neutrophils, and macrophages in their lungs at day 3-post infection. At days 5 and 7, the numbers of leukocytes were similar in WT and NKLAM^-/-^ mice. As shown in Fig 6B, using hematoxylin and eosin (H&E) staining, we found that the degree of inflammation was similar in WT and NKLAM^-/-^ mice at day 7 post-infection. This observation agrees with the flow cytometry studies (Fig 6A). The lungs of both genotypes showed evidence of leukocyte accumulation in the airways (white arrows). However, at day 14 post-infection, the lungs of NKLAM^-/-^ mice had diminished inflammation as compared to WT mice. There was evidence of perivascular leukocyte accumulation (black arrow) in WT but not NKLAM^-/-^ lungs at day 14. These results suggest that NKLAM^-/-^ mice may resolve inflammation earlier than WT mice and that NKLAM may play a role in controlling leukocyte migration into the lung during the early stages of infection.

### Lack of NKLAM is associated with attenuated autophagic flux and less expression of autophagy-related proteins LC3 and p62/SQSTM1

**Fig 7:**
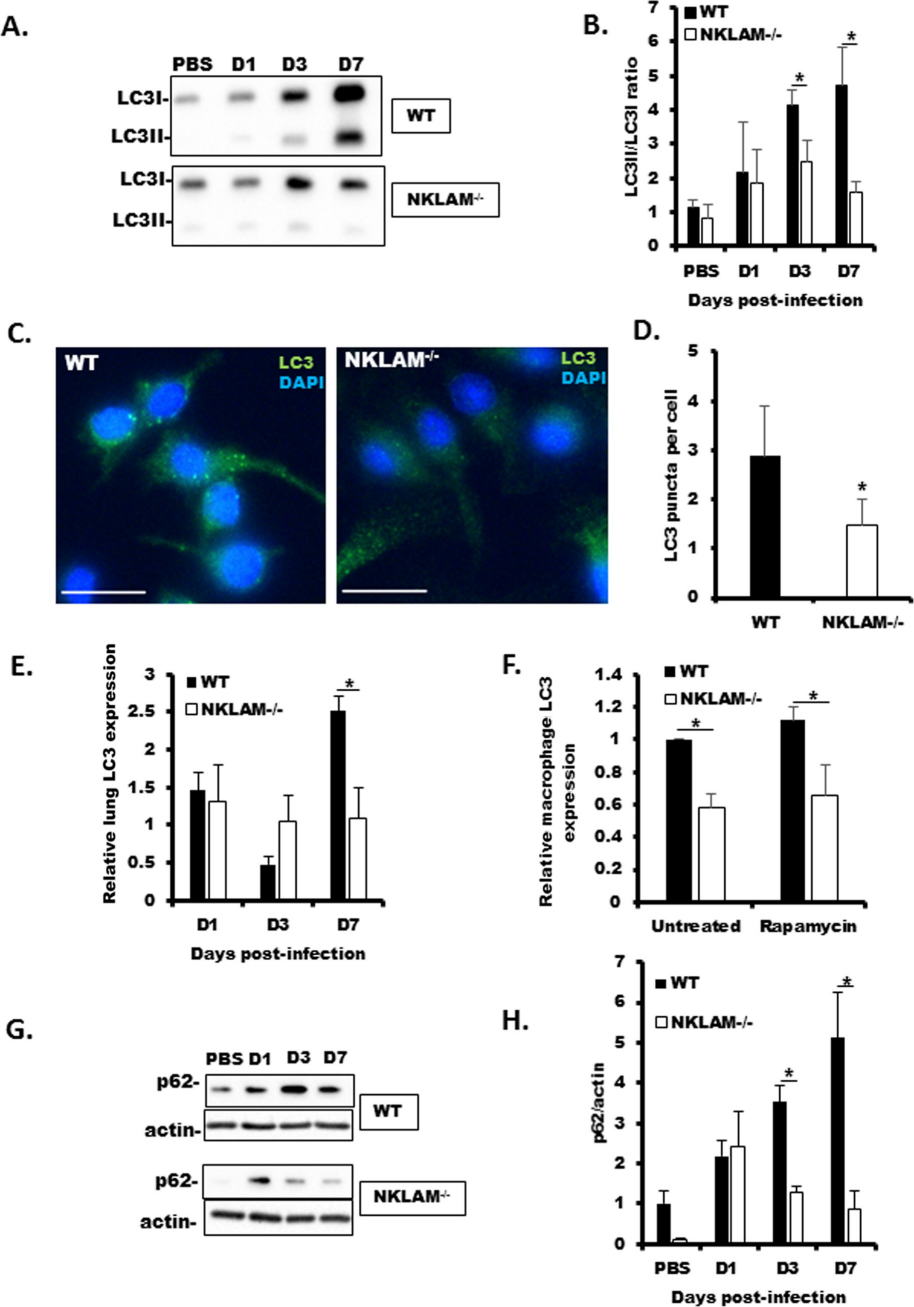
Autophagy marker LC3 expression is less in NKLAM^-/-^ mice and BMDM. WT and NKLAM^-/-^ mice were infected with 500 pfu SeV/gram body weight and the conversion of LC3I to LC3II was determined by Western blot. A representative blot is shown in A. The ratio of LC3II/LC3I is shown graphically in B. n = 3 mice per group; * p < 0.05; comparing NKLAM^-/-^ and WT mice. C) WT and NKLAM^-/-^ BMDM were treated with 5 μg/ml rapamycin for 18h and stained for LC3 (green). Nuclei were counterstained with DAPI (blue). The number of LC3 puncta/cell was quantified using ImageJ. At least 250 cells were counted for each condition (D). White bar is 15 μm; * p ≤ 0.05. Data represent 3 independent experiments. E) RNA isolated from lung homogenate from SeV-infected lungs was used to determine LC3 expression by qPCR. F) WT and NKLAM^-/-^ BMDM were treated with 5 μg/ml rapamycin for 18h. LC3 protein expression was determined by Western blot and normalized to total protein using TCE. WT untreated values were set to 1. Data represent 3 separate experiments. SeV-infected lung homogenate was used to determine p62 expression by Western blot (G) and by qPCR (H). mRNA levels (mean ± SD) are expressed relative to PBS-treated mice; n = 3 mice per group; * p ≤ 0.05; comparing NKLAM^-/-^ and WT mice.

The attenuated viral replication in NKLAM^-/-^ mouse lung and tracheal homogenate (Fig 3) was unexpected. As some members of the paramyxoviruses require autophagy to successfully replicate [32], our next set of experiments was designed to examine autophagic flux in WT and NKLAM^-/-^ mice. As shown Fig 7A and 7B, the lack of NKLAM is associated with reduced in autophagic flux in NKLAM^-/-^ mouse lungs, as the conversion of LC3I to LC3II is significantly less in NKLAM^-/-^ than WT mice at days 3 and 7. Autophagy is associated with translocation of LC3 to the autophagosome, and can be characterized by punctate cytoplasmic staining. Our immunofluorescence studies demonstrated that NKLAM^-/-^ BMDM formed significantly less LC3 puncta in response to rapamycin inducer (Fig 7C and 7D). We also found that total LC3 expression at days 7 and 14 post-SeV infection was less in NKLAM^-/-^ mouse lungs, as determined by qPCR (Fig 7E). Total LC3 protein was also less in NKLAM^-/-^ BMDM (Fig 7F). Additionally, the expression of p62 in lung homogenate was lower in NKLAM^-/-^ mice during SeV infection (Fig 7G and 7H). Collectively, our results suggest that the lack of NKLAM is associated with a deleterious effect on autophagic flux and the expression of key autophagy-related proteins.

### High-dose SeV survival studies

**Fig 8:**
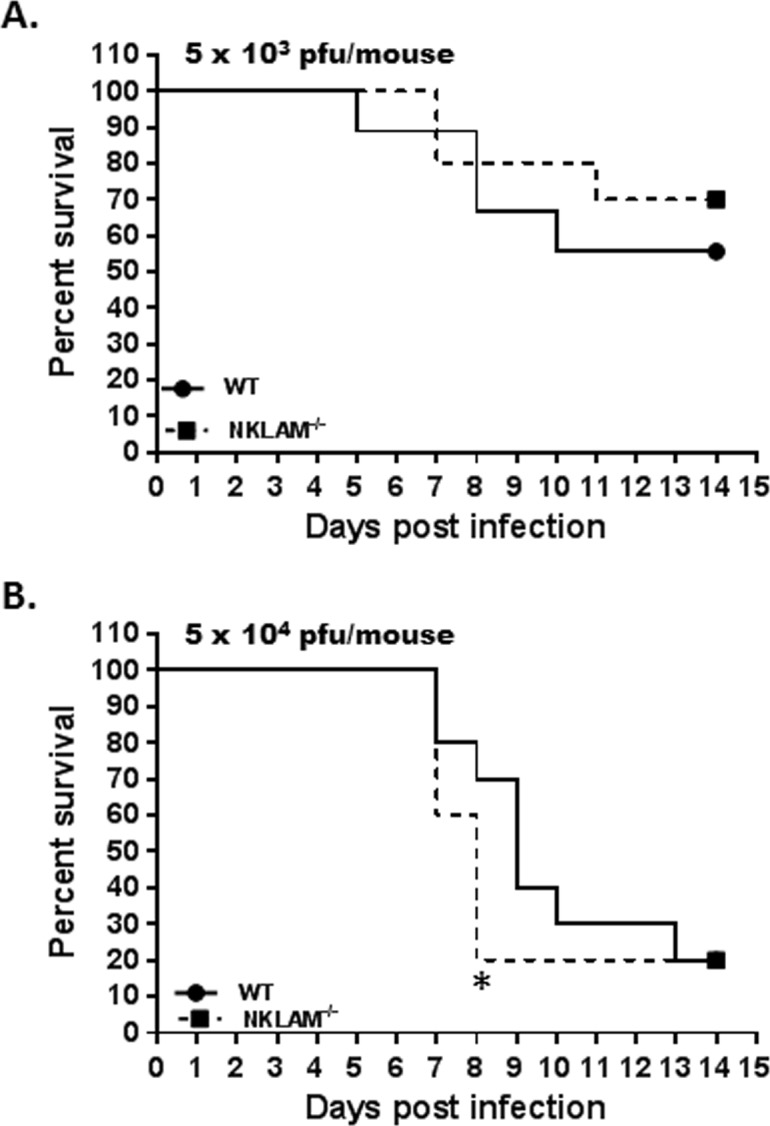
High-dose SeV infection is associated with a faster mortality rate in NKLAM^-/-^ mice. Wild type and NKLAM^-/-^ mice were infected with 5 x 10^3^ (A) or 5 x 10^4^ (B) pfu SeV/gram body weight and monitored for signs of distress for 14 days. * p < 0.05 at day 8; Log-rank test for significance; n = 10 mice per group.

WT and NKLAM^-/-^ mice were nasally infected with either 5 x 10^3^ (Fig 8A) or 5 x 10^4^ (Fig 8B) pfu/gram body weight of SeV. Both doses have been shown to induce lethality in previous studies [33]. At 5 x 10^3^ SeV pfu/gram body weight, both genotypes lost mice during the study period. The NKLAM^-/-^ group fared slightly better with 70% of the mice surviving as compared to 56% in the WT group; however, the difference did not reach statistical significance. At 5 x 10^4^ pfu/gram body weight, the mortality rate increased drastically. At day 8, only 20% of the NKLAM^-/-^ group had survived. In comparison, 70% of the WT group were still alive at day 8. There was a significant difference in survival between WT and NKLAM^-/-^ only at day 8 (p = 0.04). During the study period from day 8 to 14, several more of the WT mice succumbed to SeV infection and the final survival percentage was 20% for both WT and NKLAM^-/-^ mice. Collectively, there was no significantly difference in survival overall.

## Discussion

Our current study describes a novel role for RBR E3 ubiquitin ligase NKLAM as a component of the innate immune response against viral infection. Our data show that NKLAM is central to the production of inflammatory mediators, as mice lacking NKLAM have significantly lower cytokine and chemokine production compared to WT mice.

A common symptom of colonization with infectious pathogens (e.g. bacteria, viruses) is weight loss. Kamperschroer et al. demonstrated that during infection with lymphocytic choriomeningitis virus, the production of pro-inflammatory cytokines, such as IFNγ, IL-1 and IL-6 was involved in wasting disease and that wasting is an effect of the host’s immune response towards the virus and not viral replication [34]. Our current study echoes this point as the greater weight loss in WT mice corresponded to higher levels of pro-inflammatory cytokines, namely IL-6 and IFNγ at day 7 post-infection (Fig 5 and Table 1). A hallmark of pulmonary infection is the recruitment of leukocytes from circulation to the lung. As the infection progresses, cytokine/chemokine production by lung epithelial cells and resident macrophages induces the directed migration of leukocytes into the lung. In our model, we found that WT mice had higher numbers of neutrophils and macrophages in the lung at day 3 post-infection (Fig 6). The numbers for both cell types normalized by day 5. One explanation for the increased leukocytes in WT lungs is the higher level of chemokine production compared to NKLAM^-/-^ mice. Most of the cytokines tested were significantly increased in WT lungs at day 7 post-infection (e.g. MCP-1, MIP-2, RANTES); however, KC was significantly increased in WT lungs at day 3 (Fig 5 and Table 1). KC is the murine homolog to human IL-8 and thus is a powerful neutrophil chemoattractant [35]. A number of cytokines were similar between SeV-infected WT and NKLAM^-/-^ mice (S1 Table). There was also a subset of cytokines that were expressed at greater levels in NKLAM^-/-^ mice as compared to WT mice (S2 Table); suggesting that NKLAM may have positive and negative effects on cytokine expression. Collectively, our results suggest that NKLAM may, in part, regulate directed migration of leukocyte subsets during infection, a process critical to successful pathogen eradication.

Cytokines are critical regulators of inflammation and serve to recruit immune cells to sites of infection and stimulate those cells to defend the host. Transcription factors from the STAT family, in addition to NFκB, are important regulators of cytokine production. Previous studies from our laboratory place NKLAM as an important regulator that promotes the transcriptional activation of NFκB and STAT1 [11, 36]. In our model of SeV infection, STAT1 and NFκB p65 phosphorylation as well as STAT1 protein expression are lower in NKLAM^-/-^ mice (Fig 4). We believe this dysregulation of immune transcription factor activation is partly responsible for the diminished global cytokine expression phenotype in NKLAM^-/-^ mice. While ubiquitination has been shown to play an important role in immune signaling, studies that define a role specifically for RBR ubiquitin ligases are few. One such study from Tokunaga et al. demonstrates that RBR family members HOIL-1L and HOIP polyubiquitinate NEMO, a component of the canonical NFκB signaling pathway, which induces NFκB activation [37]. Thus, research into the role of RBR ligases as facilitators of inflammatory cytokine expression merits further study.

An alternative hypothesis to consider is that the lower cytokine production in NKLAM^-/-^ mice is the result of attenuated viral presence and not a direct effect of NKLAM. This indeed is a possibility; however, our data suggest that it is the lack of NKLAM that contributes to lower viral load. Additionally, NKLAM has been shown to directly affect the transcriptional activity of both STAT1 [36] and NFκB [11].

Overall, the phenotype of NKLAM^-/-^ mice within the context of this study is one of innate immune system deficiency; thus, the lower SeV genome expression (viral load) in NKLAM^-/-^ mouse lungs was unexpected. A similar result was observed in a recent study from Bharaj et al. that demonstrated Ebola replication was dependent on an E3 ubiquitin ligase named TRIM6 [38]. The authors found that Ebola protein VP35 hijacks TRIM6 to promote Ebola replication. Viruses of the paramyxovirus family, including Sendai and human respiratory syncytial virus, require autophagy to replicate [39, 40]. Current studies suggest paramyxovirus-induced autophagy may promote replication through cell-cell fusion [41] or inhibition of apoptosis through maintenance of mitochondrial homeostasis via mitophagy [39]. Using lung homogenate, we showed that NKLAM^-/-^ mice had a defect in conversion of LC3I to LC3II; suggesting the lack of NKLAM is associated with defects in autophagic flux (Fig 7A and 7B). Hou et al. demonstrated that siRNA knockdown of key autophagy-related proteins ATG7 or LC3 resulted in significant reduction of replication of Avian metapneumovirus, a member of the paramyxovirus family [42]. Additionally, siRNA knockdown of p62 has also been shown to inhibit paramyxoviral replication [43]. Our data demonstrate that NKLAM^-/-^ mice express significantly less LC3 and p62 than WT mice and this reduced expression of key autophagy proteins may contribute to the observed reduced SeV genome expression. We analyzed 1000 bases of the mouse LC3 promoter region using the online transcription factor binding site database PROMO and found consensus sites for both STAT1 and NFκB. Additionally, an NFκB response element has been found in the p62 promoter [44]. Thus, an attractive hypothesis for the lower SeV replication in NKLAM^-/-^ mice is one where the lack of NKLAM, and subsequent dysregulation of STAT1 and/or NFκB function, leads to attenuated LC3 and/or p62 expression and diminished SeV replication. Precisely how LC3 and p62 play a role in paramyxovirus replication is not well understood. Further studies that define the precise role of NKLAM in the regulation of LC3 or autophagic flux are warranted.

With respect to our survival studies, the lack of NKLAM was slightly, albeit not statistically, beneficial when the intermediate dose of SeV (5 x 10^3^ pfu/gram body weight) was used. However, when NKLAM^-/-^ mice were infected with the highest SeV dose (5 x 10^4^ pfu/gram body weight), the survival rate for NKLAM^-/-^ mice at day 8 was only 20% versus 70% in the WT population (Fig 8). Thus, when NKLAM^-/-^ mice were placed in a high pathogen pressure scenario (high dose SeV), their immune system may have been overwhelmed and the lack of a normal immune response compromised the animals and led to a greater mortality.

Based on the data present here, we propose that RBR E3 ubiquitin ligase NKLAM/RNF19b plays a significant role in directing the immune response to viral pathogens by regulating the production of key inflammatory cytokines that are critical for successful pathogen eradication.

## Supporting information

S1 Fig Flow cytometry gating strategy. (PDF)

S1 Table Cytokine array: Genes that were expressed to a greater degree in NKLAM^-/-^ mice as compared to WT mice. (PDF)

S2 Table Cytokine array: Genes that were similarly expressed in WT and NKLAM^-/-^ mice. (PDF)
