# Supplementary figures and images for: Mice deficient in NKLAM have attenuated inflammatory cytokine production in a Sendai virus pneumonia model
Source: PLoS One. 2019 Sep 20;14(9):e0222802. doi: 10.1371/journal.pone.0222802 (PMC6754162; doi:10.1371/journal.pone.0222802)

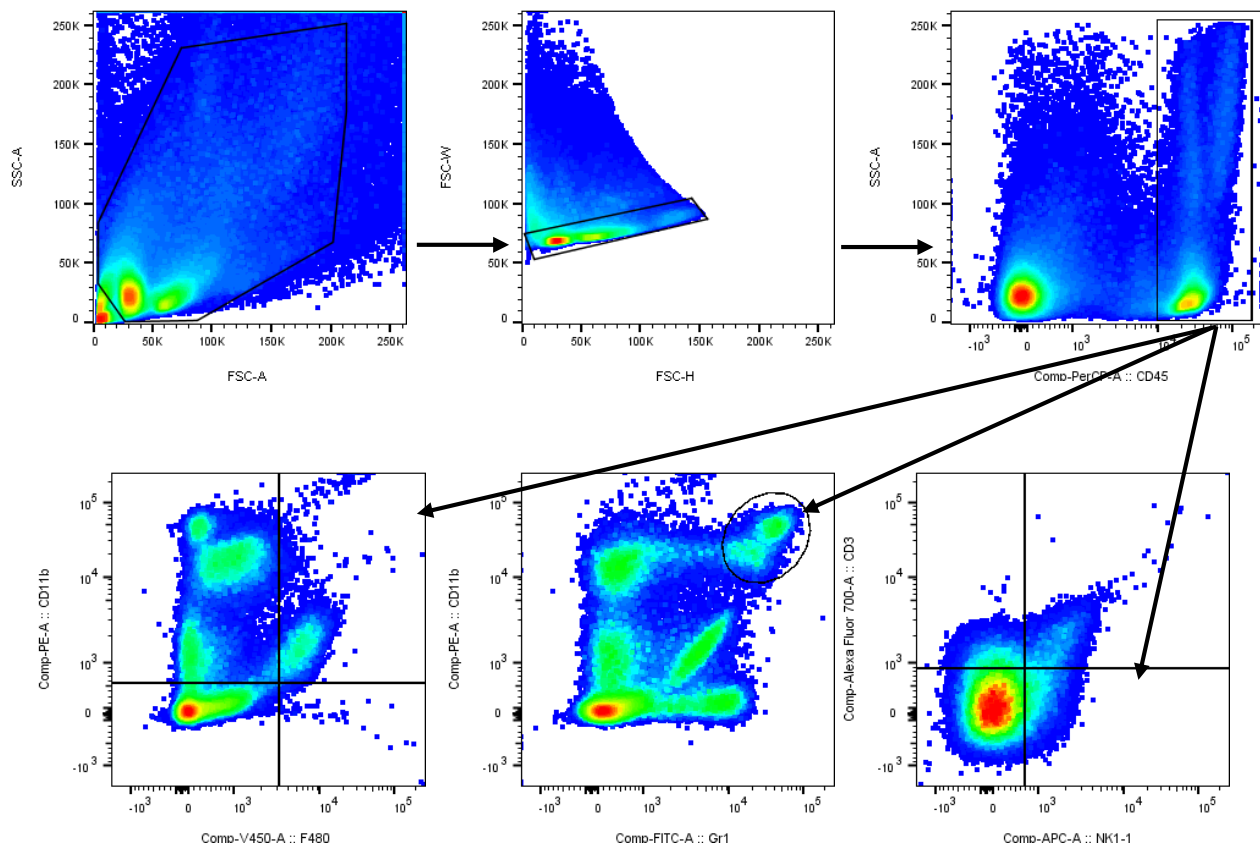

Supplement: S1 Fig — (PDF) [file pone.0222802.s001.pdf]
