# Supplementary material for: Mice deficient in NKLAM have attenuated inflammatory cytokine production in a Sendai virus pneumonia model
Source: PLoS One. 2019 Sep 20;14(9):e0222802. doi: 10.1371/journal.pone.0222802 (PMC6754162; doi:10.1371/journal.pone.0222802)
Supplement: S1 Table — (PDF) [file pone.0222802.s002.pdf]

Day 3

Cytokine:	WT	NKLAM$^{-/-}$	fold
CXCL16	44473	36662	1.2
L-selectin	77170	65507	1.2
MCP-5	11894	10755	1.1
IGFBP-3	32544	28856	1.1
LIX	170135	160639	1.1
P-selectin	35386	35501	1.0
Eotaxin-2	8307	8509	1.0
TARC	21272	21298	1.0
TNFRII	28586	31801	0.9
fractalkine	34985	43867	0.8
MIP1g	113866	137736	0.8
TNFR1	114851	147605	0.8
IGFBP-6	30407	42806	0.7
CD40	6438	8846	0.7
VCAM-1	57522	85128	0.7
IL-4	21098	27345	0.8
CD30	n.d.	n.d.	-

Day 7

Cytokine:	WT	NKLAM$^{-/-}$	fold
TARC	49733	38736	1.3
TNFRII	85467	69941	1.2
CXCL16	45493	39451	1.2
TIMP-1	21305	19039	1.1
TNFa	18981	16652	1.1
MIP1g	276926	250696	1.1
IL-4	20912	20709	1.0
MIP3a	28497	29648	1.0
leptin R	34784	34902	1.0
IGFBP-3	55817	56212	1.0
L-selectin	108593	109273	1.0
TNFR1	247894	264430	0.9
P-selectin	45873	51050	0.9
LIX	195140	223512	0.9
fractalkine	38418	50044	0.8
VEGF-a	8586	12503	0.7
VCAM-1	99661	136289	0.7
CD30	n.d.	n.d.	-
MIG	n.d.	n.d.	-
