# Supplementary material for: Mice deficient in NKLAM have attenuated inflammatory cytokine production in a Sendai virus pneumonia model
Source: PLoS One. 2019 Sep 20;14(9):e0222802. doi: 10.1371/journal.pone.0222802 (PMC6754162; doi:10.1371/journal.pone.0222802)
Supplement: S2 Table — (PDF) [file pone.0222802.s003.pdf]

Day 3

Cytokine:	WT	NKLAM$^{-/-}$	fold
TARC	865	8536	9.9
VEGF-a	2036	13408	6.6
lymphotactin	2945	11462	3.9
IGFBP-5	2048	6366	3.1
PF-4	17236	44855	2.6
leptin R	12662	28672	2.3
TPO	14841	25079	1.7
TIMP-1	7832	12709	1.6
IL-12 p70	9564	15798	1.7
M-CSF	11192	18323	1.6
MIP3a	13349	21080	1.6
IL-1b	n.d.	5824	-
TNFa	n.d.	10839	-
MIP3b	n.d.	4077	-
Fas ligand	n.d.	3639	-
MIG	n.d.	808	-

Day 7

Cytokine:	WT	NKLAM$^{-/-}$	fold
IL-12 p70	698	6087	8.7
Fas ligand	570	2360	4.1
M-CSF	4488	17670	3.9
lymphotactin	1592	5484	3.4
IL-1b	1633	4069	2.5
Eotaxin-2	4895	11926	2.4
MCP-5	3627	8084	2.2
TPO	15576	32283	2.1
IGFBP-6	23951	49575	2.1
CD40	7155	11779	1.6
MIP3b	n.d.	1746	-
IGFBP-5	n.d.	13394	-
PF-4	n.d.	40059	-
TARC	n.d.	5672	-
